# The double edged interferon riddle in COVID-19 pathogenesis

**DOI:** 10.1186/s13054-020-03337-z

**Published:** 2020-11-01

**Authors:** Rahul Gupta

**Affiliations:** Kolkata, India

Dear Editor,

In their recent article [1], Jalkanen et al. discuss about the prospective usage of interferon beta 1 in managing COVID-19 and substantiating usage of intravenous route of administration over subcutaneous route. I would like to humbly add some views to it: there has been two varying reported type I interferon responses in COVID-19 pathogenesis [2]: one stating the suppression of host antiviral type I interferons (IFNs) and interferon stimulated genes (ISGs) and other stating increased expression of different ISGs, with further inductions of chemokines and cytokines [2].

The viral Nsps (particularly Nsp1) and the ORFs (particularly ORF 6) are known to antagonise the host antiviral IFNs initially by suppressing/delaying their expressions, leading to viral persistence and propagating inflammations. Hence, neither type I IFN nor type III IFN, which are known hard-wired for providing antiviral immunity, was activated in early stages of COVID-19. However, SARS-CoV-2 at 2 days post-infection (dpi), induced ISGs having antiviral action (Rsad2, Ifit, Mx2, Oas3, etc.) and at 7dpi, ISGs having potentiating IFN mediated inflammatory signalling (Ifihi,Irf7,Stat1,Ifnar1/2,Tyk2,etc.) [3]. As the disease progresses towards severity, the IFNs exacerbate the pathophysiology with specific inflammatory signatures [2]. Hence, cellular response to type 1 IFN (thru ISGs) towards later stages of infection is immunopathogenic.

Neutrophils provide the first line of innate immune defence. Neutrophil attracting chemokines (CXCL1, CXCL2, CXCL8, S100A9) and cognate receptor (CXCR2) were found to be activated in early stages (1–3 dpi) [3]. COVID-19 is manifested with necrophilia having high neutrophil-to-lymphocyte ratio. Type 1 IFNs are known to inhibit neutrophil migration by downregulating neutrophil chemoattractants production (CXCL1/2) [4]. Other than phagocytosis, neutrophils have another capacity to contain pathogens, by forming neutrophil extracellular traps (NETs). NETs are mesh-like structures of DNA and proteins from degrading neutrophils (by neutrophil elastase) which entrap pathogens. Interestingly against *leishmania*, IFNAR^−/−^ mice showed enhanced neutrophil elastase activity, with better infiltrations. Aberrant production of NETs have been known to cause severe COVID-like pathophysiologies—thrombosis, lung damage, ARDS, multiorgan damage, etc. [5]. Indeed, severe COVID-19 patients reported of higher amount of NETosis remnants like cell-free DNA, myeloperoxidase-DNA and citrullinated histone H3 [5]. These molecules further propagate inflammation by inducing IL-1β production thru inflammasome activation.

The initial type 1IFN suppression could lead to enhanced infiltration of neutrophils, NET formation and ensuing pathophysiologies. Early administration of IFNβ has proved beneficial [1, 2]; hence, the “double edged sword” be tried prudently with respect to time and dosage.

## Data Availability

Literature survey.
